# Interactive effects of pests increase seed yield

**DOI:** 10.1002/ece3.2003

**Published:** 2016-02-29

**Authors:** Vesna Gagic, Laura GA Riggi, Barbara Ekbom, Gerard Malsher, Adrien Rusch, Riccardo Bommarco

**Affiliations:** ^1^ Department of EcologySwedish University of Agricultural SciencesSE‐75007UppsalaSweden; ^2^ INRAISVVUMR1065 Santé et Agroécologie du VignobleF‐33883Villenave d'OrnonFrance

**Keywords:** Herbivore, oilseed rape, plant compensation, plant damage, pollen beetle, weevils

## Abstract

Loss in seed yield and therefore decrease in plant fitness due to simultaneous attacks by multiple herbivores is not necessarily additive, as demonstrated in evolutionary studies on wild plants. However, it is not clear how this transfers to crop plants that grow in very different conditions compared to wild plants. Nevertheless, loss in crop seed yield caused by any single pest is most often studied in isolation although crop plants are attacked by many pests that can cause substantial yield losses. This is especially important for crops able to compensate and even overcompensate for the damage. We investigated the interactive impacts on crop yield of four insect pests attacking different plant parts at different times during the cropping season. In 15 oilseed rape fields in Sweden, we estimated the damage caused by seed and stem weevils, pollen beetles, and pod midges. Pest pressure varied drastically among fields with very low correlation among pests, allowing us to explore interactive impacts on yield from attacks by multiple species. The plant damage caused by each pest species individually had, as expected, either no, or a negative impact on seed yield and the strongest negative effect was caused by pollen beetles. However, seed yield increased when plant damage caused by both seed and stem weevils was high, presumably due to the joint plant compensatory reaction to insect attack leading to overcompensation. Hence, attacks by several pests can change the impact on yield of individual pest species. Economic thresholds based on single species, on which pest management decisions currently rely, may therefore result in economically suboptimal choices being made and unnecessary excessive use of insecticides.

## Introduction

Crop plants are attacked by many pests that affect plant survival, growth, and reproduction and as a result influence crop yield (Oerke 2006). Herbivory by an insect typically reduces plant performance (Bigger and Marvier 1998; Kolb et al. 2007). However, a plant is often attacked by several insect species and their combined attacks might nonadditively, that is, interactively, affect biomass and seed yield. The magnitude and frequency as well as the net effect of such interactive outcomes on crop plants are not well understood.

Both nonadditive and additive negative effects of multiple herbivorous insects on plant fitness have been reported. Nonadditive effects are generally negative and either greater (more negative) or lower (less negative) than expected when either of the insect species feeds alone (Strauss 1991; Karban and Strauss 1993; Wise and Sacchi 1996; Hufbauer and Root 2002; Barber et al. 2012). This often occurs if one herbivore facilitates or negatively affects another herbivore species (Stephens et al. 2013). In a meta‐analyses, Stephens et al. (2013) found that in one‐quarter of cases the effect of plant enemies on plant performance was nonindependent and mostly led to a smaller reduction in plant performance than that predicted when each enemy feeds alone. Additive effects of insect attacks on wild plant fitness are also typically negative (Maron 1998; Puliafico et al. 2008; Irwin and Brody 2011; Stephens et al. 2013). However, the majority of these studies are evolutionary and have been conducted on wild plants contrasting pairwise with diffuse plant**–**herbivore coevolution, while current agro‐ecological research is almost exclusively focused on individual pests. Little is known about interactive effects of insect pests on crop plant fitness and thereby on crop seed yield, although crops differ from wild plants in several ways. Herbivores damage crops more than wild plants, presumably due to genetic changes in plants and herbivores following domestication (Morris et al. 2007). Artificial selection for palatability for humans may make crop plants more vulnerable to other herbivores, while increased nitrogen input can make them more prone to pest attack. On the other hand, plants coexist closely in crop fields and their exposure to volatile organic compounds from neighboring plants may ‘prime' them to respond more efficiently to herbivore attacks once they themselves are attacked (Heil and Kost 2006). Additionally, anthropogenic inputs of water and nutrients to the crop can affect the ability of a plant to compensate for damage by regrowth following herbivory (Levine and Paige 2004; Wise and Abrahamson 2007). Given the increasing evidence for nonadditive effects of multiple herbivores on wild plants, it might be problematic to predict crop losses based on information on attacks of single pest species. If interactive effects among insect pests are common in crop fields, wrong pest management decisions can be taken, as they are based on economic thresholds that are almost entirely derived from studies of single, or several closely related pest species expected to have similar and additive effect.

Combined effects of multiple, distantly related insect pests have mostly been investigated when there is facilitation of one pest by another. For example, the economic threshold for the seed weevil (*Ceutorhynchus assimilis* Payk., Curculionidae, Coleoptera) in oilseed rape is lower when the pod midge (*Dasineura brassicae* Winn., Cecidomyiidae, Diptera) is present. The midge uses the seed weevil feeding punctures and larval exit holes to infest the plant (Alford et al. 2003). Furthermore, Dangles et al. (2009) found that herbivore insect diversity increases potato yield loss, but the effect of insect density and time of the attack was not tested. However, plants with high levels of early herbivory can suffer less damage by late herbivores due to changes in plant traits (Barber et al. 2012). According to a recent meta‐analysis, plant performance should be independently reduced by natural enemies sharing a host plant, except in cases where enemies attack the same plant part concurrently (direct interactions) or attack plant reproductive structures. In such cases, multiple enemies should lead to a smaller reduction in performance than that predicted from each enemy alone (Stephens et al. 2013). Moreover, the net effect of combined insect attack may vary depending on the damage levels of each herbivore species (Lerin 1988; Pilson 1996) and depends on the plant's ability to compensate for damages. Several instances of overcompensation for pest‐induced damages have been reported for pairwise plant‐insect interactions (Belsky 1986; Crawley 1987; Owen 1980; Paige and Whitham 1987; Williams 2010), but little is known about whether plants can overcompensate for damage caused by multiple herbivores.

Here, we used oilseed rape as a model system because it is an important crop for proteins and oil and it has high compensatory abilities for insect damage (Free and Williams 1978, 1979; Williams and Free 1979). Nevertheless, yield losses of 70–80% have been recorded in oilseed rape not treated with insecticides (Nilsson 1987; Hansen 2003). Together with the seed weevil and the pod midge, the most common insect pests on winter oilseed rape in northern Europe are the pollen beetle (*Meligethes aeneus* F., Nitidulidae, Coleoptera) and the cabbage stem weevil (*C. pallidactylus* Marsh., Curculionidae, Coleoptera). These four species attack either different plant parts or during different stages of the plant's development (Alford et al. 2003). Pollen beetle infestation can result in blind stalks that prevent pod development, while stem weevils cause distortion of stem tissue and loss of plant vigor. Pod midge and seed weevil infestations often result in considerable seed loss due either to the direct effect of larvae feeding on seeds, or due to the development of yellow pods that split open to release midge larvae, simultaneously dropping seeds. (Williams 2010). The magnitude of the negative effects of these four pest species on crop yield differs greatly, with pollen beetles being identified as the major oilseed rape pests, while stem weevils rarely cause economically significant losses (Alford et al. 2003). Pest infestations can also trigger compensation in oilseed rape. For example, bud abortion can lead to the production of new racemes and buds, and seed loss can be compensated by higher seed weight (Free and Williams 1978, 1979; Williams and Free 1979). Given the difference in the magnitude of the negative pest effects on yield and in the plant compensatory mechanisms, the net effect of combined pest attack on seed yield is hard to predict.

We used fifteen oilseed rape fields to investigate how plant damage caused by four insect pest species (brassica pod midge, seed weevil, stem weevil, and pollen beetle) in concert affect oilseed rape seed yield. We focused on the interactive effects of plant damage caused by insects on seed yield and tested the presence and direction of these effects.

## Materials and Methods

### Study design

The study was carried out in 2013, in 15 conventionally managed winter oilseed rape fields in the Swedish province Västergötland. The land‐use in the region is characterized by arable land (19.42%), woodlands (20%) and pastures/meadows (2.53%) (http://statistik.sjv.se/, data from 2013), while the rest of the area is covered mainly by built‐up areas, lakes, and waterways. To ensure among‐field differences in pest colonization and subsequent pest pressure, the fields were chosen along a gradient of agricultural intensity measured as percentage arable land in a circular landscape sector of 1.5 km diameter encompassing the field (Zaller et al. 2008a). Percentage arable land in the landscape sectors varied between 38% and 93.62%. Due to a wet autumn in 2012 and harsh winter conditions, the oilseed rape area in the landscape sectors had decreased on average by 25% in 2013 compared to 2012. This allowed us to conduct our study with unusually high pest pressures as the pests converged on the remaining fields. Insect pest abundances per plant exceeded the economic thresholds in many fields. Especially high were attacks by pollen beetles during the most susceptible green bud stage and by seed weevils (60% and 73% fields, respectively, exceeded the economic thresholds).

To avoid high multicollinearity in the data and ensure that the colonization of the fields by the four pests and the subsequent damage would not strongly covary, the landscape sectors were selected so that percentage oilseed rape, grassland and woodland area in the landscape sectors are not correlated. This is because the four investigated pest species respond differently to these landscape elements. Pollen beetle and stem weevil damages to oilssed rape have been shown to be negatively related to proportion oilseed rape and positively related to proportion woodlands at a 1.5 km landscape scale, although the response of pollen beetles was much stronger (Zaller et al. 2008a). Pod midge conversely does not seem to respond to either woodland or oilseed rape percentage at 1.5 km landscape scale (Zaller et al. 2008a). Grassland meanwhile can support high overwintering populations of pollen beetles, but also of the pests' natural enemies (Rusch et al. 2013). It can positively affect rates of parasitism in pollen beetles (Rusch et al. 2011) and activity‐denisty and richness of generalist natural enemies (Purtauf et al. 2005). To verify our design we checked for multicollinearity in the data by studying the VIF (variance inflation factors) for all parameters included in the statistical models. There was no collinearity, as indicated by VIF‐values lower than 1.5 in all cases (Zuur et al. 2009).

To ensure spatial, independence selected fields were at least 3 km apart. Within each field, we selected a 40 × 70 m unsprayed area located at the edge of the field. Observations were made along two transects parallel to the field edge, 3 and 30 m into the field. In each transect, we estimated insect damage and seed yield per plant, as well as plant density and weed cover per square meter at five sample points 10 m apart and a minimum of 10 m away from the sprayed area. The sampled field edge was next to a grassy strip (of minimum 2 m width) in all fields.

### Plant damage sampling

#### Pollen beetles

Adult pollen beetles cause most damage to oilseed rape plants when they feed on pollen in the buds early in the season, affecting developing racemes and causing bud abortion (Alford et al. 2003). To assess pollen beetle damage, we calculated the percentage of podless stalks in relation to total number of pods on 10 randomly chosen plants per sampling point (100 plants in total per field) at GS (growth stages) 77–79 (70–100% pods having reached final size, cf. Lancashire et al. 1991). Since podless stalks can be caused by other factors, we correlated percent podless stalks with the mean pollen beetle density and found high correlation (Pearson's product‐moment correlation = 0.35, *P* < 0.001). Pollen beetle density was estimated using data from visual counts of adults. Pollen beetle counts were conducted weekly on ten randomly chosen plants per sampling point, three times in total during the season from oilseed rape green bud stage until flowering.

#### Stem weevils

Stem weevil larvae feed gregariously for 3–5 weeks and form extensive galleries in the oilseed rape stems that cause distortion of tissue and loss of plant vigor (Alford et al. 2003). To assess damage by stem weevil larvae, we dissected 10 randomly chosen plant stems per sampling point at GS 64‐67 (oilseed rape flowering, Zaller et al. 2008b). We calculated the percentage of stem damage (including zeros) as the length of the damaged part (stem length exhibiting visible signs of weevil herbivory) divided by the total stem length. Additionally, we measured the diameter of the main stem of each sampled plant.

#### Seed weevils

Seed weevil adults typically deposit one egg per pod in which the larva usually destroys an average of five seeds (Alford et al. 2003). To assess the level of seed weevil damage, 10 pods were randomly selected at each sampling point (GS 80‐81, beginning of ripening) and the percentage of pods with emergence holes was used as a measure of seed weevil damage.

#### Brassica pod midge

The brassica pod midge causes most damage to the plant when the larvae feed on the inner walls of the pods late in the season (Alford et al. 2003). To assess damage by brassica pod midge larvae (GS 77‐79) we picked ten randomly chosen plants per sampling point and estimated the percentage of premature, yellow and split pods. (Zaller et al. 2008b).

### Seed yield

Three components of seed yield in oilseed rape were assessed: pod number per plant, seed number per pod, and seed weight. To measure total seed yield (total seed weight per plant) we calculated the number of healthy, fully developed pods per plant multiplied by the average number of seeds per pod and the average seed weight. Number of seeds per pod was counted on 20 pods randomly chosen from terminal raceme from five plants at each sampling plot. We measured seed weight per sampling point by selecting and weighing three subsamples of 100 seeds from randomly chosen pods. The farmers were interviewed and information about crop cultivar and management practices such as fertilization and yield was collected. To check how our estimation of seed yield (estimated per plant) correlated with the farmers' estimates of yield (per area), we multiplied our seed yield measurements with the estimates of plant numbers per square meter to get an area based measure (averaged per field). We found a high correlation (Pearson's product‐moment correlation = 0.74, *P* = 0.01).

### Statistical analyses

For the analyses of total seed yield (total seed weight per plant) and its individual components (1) number of healthy, fully developed pods; (2) average seed number per pod; (3) average seed weight, we used mixed effects models with edge effect (distance from edge: 3 m and 30 m) nested within fields as random factors. As predictors, we used percentage podless stalks (as a proxy for pollen beetle damage), percentage stem damage (as a proxy for stem weevil damage), percentage pods with seed weevil exit holes (as a proxy for seed weevil damage) and percentage yellow, split pods (as a proxy for pod midge damage) as well as their pairwise interactions.

We used multimodel inference (Burnham and Anderson 2002) to identify which of the predictors were the main determinants of yield. This approach allows for comparisons among multiple models with different combinations of predictors, and the identification of the most parsimonious model according to the criterion used. To rank the models, we used the second order Akaike information criterion corrected for a small sample size (AICc, Hurvich and Tsai 1989). For each model, we calculated the Akaike weight (wi) and used the model averaging procedure of selected models that had ∆ wi <2. If only one model was selected with this criterion, we did not use a model averaging procedure and we presented the top ranked model. All variables were standardized (to a mean of zero and a standard deviation of one) beforehand to reduce collinearity, improve model convergence and to make effect sizes easier to compare and interpret (Zuur et al. 2009; Schielzeth 2010). Due to the small sample size (*n* = 15), we restricted the number of parameters in the alternative models to four and the main effects were always in the model if interaction was present. Because there already exists solid evidence that pollen beetle damage is important for determining yield (Williams and Free 1979; Zaller et al. 2008b), we included this variable in all models. Alternative models contained two additional predictors with or without interactions yielding 13 alternative models that entered the analyses. The global model residuals were visually inspected for normality of errors and heteroscedasticity. Power or exponential variance function structure was used to improve model residuals (Pinheiro and Bates 2000) in the models for seed yield and mean seed weight. We used spline correlogram and we found no spatial autocorrelation in the model residuals. Additionally, in a preliminary analyses we found no large effect of alternative variables: weed cover, N fertilization, tiller density and cultivar on seed yield (Fig. S1), so these variables were not included in the main analysis. To make sure that the direction of causality was the one we expected, we checked whether stem weevils attack more vigorous plants, as previously reported (Dechert and Ulber 2004). We found no strong relationship between the stem weevil damage (cm) and the height or diameter of the main stem (Fig. S2). All analyses were done in R 3.1.0 (2014‐04‐10) using packages “nlme” (Pinheiro et al. 2014), “ncf” (Bjornstad 2013), and “MuMIn” (Barton 2014).

## Results

### Seed yield

**Figure 1:**
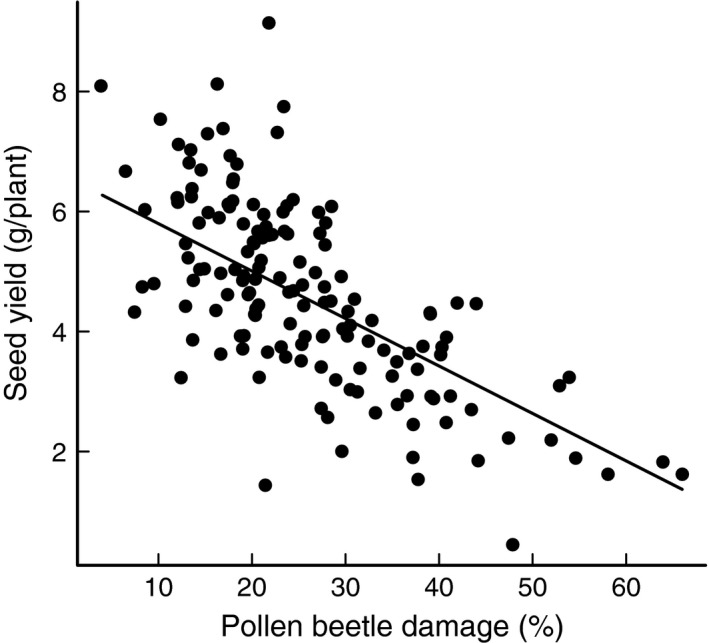
Model predictions for the relationship between oilseed rape seed yield and pollen beetle damage.

**Figure 2:**
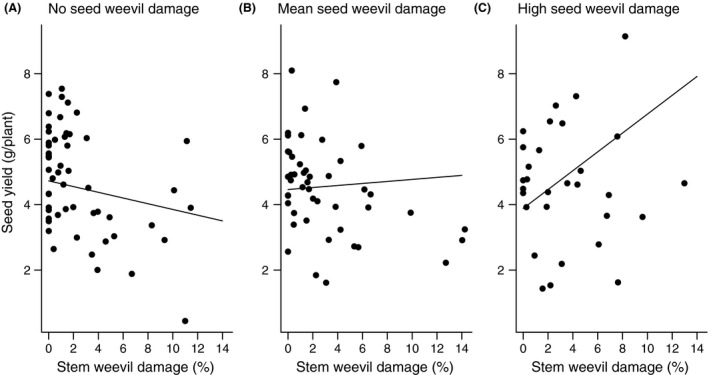
Damage to oilseed rape caused by seed and stem weevils. Model predictions for the interactive effects of stem damage at (A) no (0%), (B) mean (12.6%, full data) and (C) and high (60% < *x* > 20%, predicted regression line at 40% damage by seed weevil) levels of the seed weevil damage on seed yield. Thus, stem weevils have negative effect on seed yield when there is no damage caused by seed weevils, but when the damage by seed weevil increases the effect of stem weevils on seed yield changes from negative to positive.

Plant damage and yield varied considerably among the 15 fields (Table 1). The highest ranked model for seed yield contained the interaction between stem and seed weevil damage in addition to the main effect of pollen beetle damage, and was the only selected model with ∆*i* < 2. For this model, the AICc had a high model probability (wi = 0.87) and it was 52.9 times more likely to be better model than the second ranked model (wi = 0.02, see Table S1. fot the full model selection table). Pollen beetle damage had a negative main impact on seed yield (model estimate: −0.91 ± 0.09, *P* < 0.001, Fig. 1). Stem weevil damage (model estimate: 0.04 ± 0.11, *P* = n.s, Fig. 2A) and seed weevil damage (model estimate: 0.06 ± 0.08, *P *= n.s) had no main effect on seed yield, but in their interaction they increased yield (model estimate: 0.41 ± 0.09, *P* < 0.001, Fig. 2B and C). In particular, the model predicted high stem weevil damage to have a negative effect on yield in the absence of seed weevil damage (26% decrease in seed yield at maximum stem weevil damage), but the interaction turned increasingly positive with increasing levels of seed weevil damage (214% increase in yield at max seed and stem weevil damage). At no stem and seed weevil damage, the model predicted seed yield of 4.73 g/plant (similar to the model predictions of 4.55 g/plant at mean seed and stem weevil damage, See Table 1 for the mean, minimum and maximum values for seed and stem weevil damage).

**Table 1:** The mean, standard error (SE), median, 1st and 3rd quartile values of damge measures for four insect pests and oilseed rape yield measures across 15 fields

Damage and yield measures	Mean ± SE	Median	1st Quartile	3rd Quartile
Percentage pollen beetle damage	25.82 ± 2.28	23.40	17.95	31.18
Percentage seed weevil damage	12.60 ± 2.39	10.00	0.00	20.00
Percentage pod midge damage	5.42 ± 1.54	3.15	0.74	7.35
Percentage of stem damage	2.75 ± 0.66	1.50	0.08	4.06
Number of fully developed pods	30.81 ± 1.79	30.50	24.60	36.75
Thousand seed weight	5.70 ± 0.10	5.78	0.52	0.64
Seed number per pod	26.06 ± 0.88	25.95	22.80	29.49
Total seed yield	4.60 ± 0.13	4.52	3.62	5.73

### Individual seed yield components

Mean seed number per pod increased with seed weevil damage (model averaged coefficient, 1.09 ± 0.30, *P* < 0.001). Mean seed weight per plant was not influenced by any of the factors investigated. The best ranked model for the number of healthy and fully developed pods included the interaction between stem and seed weevil damage and the main effect of pollen beetle damage. It had a high model probability (wi = 0.757) and it was nine times more likely to be better model than the second ranked model (wi = 0.084). It was the only selected model with ∆*i* < 2. The number of healthy and fully developed pods decreased with increasing pollen beetle damage (model estimate: −5.6 ± 0.35, *P* < 0.001). Stem and seed weevil damage showed no strong main effects (model estimate: 0.29 ± 0.36, *P* = n.s and −0.40 ± 0.35, *P* = n.s, respectively), while in interaction they positively affected pod number (model estimate: 1.26 ± 0.32, *P* < 0.001). The direction of this effect was similar to the stem and seed weevil interactive effect on total seed yield.

## Discussion

By themselves, stem weevils are reported to cause little yield loss in winter oilseed rape in Northern Europe (Williams 2010), a finding confirmed in our study. Surprisingly, however, we found that although neither stem weevil nor seed weevil individually influenced seed yield, they jointly had a positive impact. When stem and seed weevils both attacked the plant, the seed yield increased more than two folds, mainly through increasing pod number. Our findings contradict predictions by Stephens et al. (2013) who argued that in cases when herbivores attack different plant parts or at different times (indirect interactions), plant performance should be independently reduced by each species. In our study, the effect on seed yield of the two herbivores (seed and stem weevils) that attack different plant parts at different times was neither additive nor negative. Furthermore, in the meta‐analysis by Stephens et al. (2013), attacks on seeds, fruits and flowers were associated with antagonistic interactions among herbivores, but in our study we did not observe any interactions among damages caused by pollen beetles, seed weevils, and pod midges that feed on plant reproductive parts.

The underlying mechanisms for the interactive effects of combined insect attacks are argued to be either interference competition among herbivores or changes in plant nutritional quality, phenology or defenses (Stephens et al. 2013). Direct interference competition between the two pests is unlikely to have occurred in our study, because stem weevils (early herbivores) and seed weevils (late herbivores) attack different plant parts and at different times. It is moreover unlikely that the observed interactive effect is due to decreased damage by later herbivores when plants suffer high early herbivory, because the stem and seed weevil damages were not correlated in our study. Instead, it is possible that early herbivore attacks ‘primed' the plants to respond more efficiently to damage by late herbivores (Heil and Kost 2006), presumably through plant compensatory mechanisms. Oilseed rape plants can compensate for high seed weevil densities by producing more pods (Tatchell 1983), but we found this only when densities of stem weevils were high. This compensatory growth can also be due to pod damage caused by adult weevils, rather than for seed loss to larvae. Compensatory growth is a common response of oilseed rape to pest injury, facilitated by redistribution of metabolites to already existing organs, or by production of new organs.

Seed weevil larvae can consume 8–15% of the seeds in a pod and reduce yield by 18% (Williams 2010). Interestingly, seed weevils did not only positively affect pod number in interaction with stem weevils, but also had an independent positive effect on seed number per pod. Although this is somewhat counter‐intuitive, a positive relationship between plant fitness (fruit set, seed yield, and seeds per fruit) and oviposition or seed predation by predispersal seed predators (i.e. where predation occurs before the seeds have been released from the parent plant) has also been documented for other plant species (Brody and Mitchell 1997; Brody and Morita 2000; Brody and Irwin 2012). This relationship may result from two mechanisms. First, oviposition by seed predators can affect the plant's reproduction. For example, Brody and Morita (2000) showed that the predispersal seed predator of *Ipomopsis aggregata* is able to induce a response in its host plant to ensure fruit set. Seed predators such as seed weevils can also locate fruits with a low probability of abortion, possibly using fruit position and phenology as cues (Östergård 2008). It is possible that the same plant trait, such as large floral displays, attracts both seed predators and pollinators (Brody and Mitchell 1997), thereby resulting in increased seed yield (Cariveau et al. 2004) even in presence of seed predation. Hence, seed predation and fruit set can be related to the same plant trait, rather than directly affecting each other. Since seed weevils affected both fruit set and seeds per fruit, all these mechanisms are possible in our study.

Pollen beetles substantially reduced seed yield, which is in accordance with a number of studies showing pollen beetles to be the main oilseed rape pests in Europe (Williams 2010 and references therein). Pollen beetle damage can induce the plant to compensate with newly produced racemes and buds, higher seed weight and lower seed number per pod (Alford et al. 2003), but this was not the case in our study. Instead, the effect of pollen beetles on the seed yield was negative, mainly because of the reduced number of healthy, developed pods. This effect of pollen beetles on seed yield was independent of the damage caused by other insects, presumably due to the absence of plant compensatory growth as a response to pollen beetle attack in our study.

Pods infested by the pod midge split open or shatter prematurely and this can result in considerable yield loss (Alford et al. 2003; Zaller et al. 2008b). However, in our study pod midge damage had little effect on seed yield, or any of the yield components (number of seeds per pod, number of healthy pods, average seed weight), although pod damage reached 12%. In contrast, Zaller et al. (2008b) found negative pod midge effects on oilseed rape yield. The difference between our findings and theirs may be explained by the different yield measures. We estimated average seed weight per plant, while Zaller et al. measured seed weight per area (ha), a measure that depends on plant density. Pod midge damage was strongly negatively correlated with plant density in our study (data not shown, see also Valantin‐Morison et al. 2007) and consequently to the area‐based yield measure (seed yield multiplied by plant density per m^2^). Hence, the scale of the yield measure, that is per plant versus per unit area, might influence conclusions of the herbivore impact on yield, since plant density can affect both pest pressure and seed yield measure. Furthermore, if one pest facilitates a second, as has been commonly reported with seed weevil and pod midge, plant performance can be reduced by their combined attack. However, this was not the case in our study, possibly because the effect of pod midge on seed yield was low or because plant compensation for the damage masked the effect.

## Conclusions

To our knowledge, this is the first study in crop fields showing that pests from different feeding guilds can not only individually affect yield through increased plant damage, but can also interactively affect yield, possibly through plant compensatiory mechanisms. Hence, the mechanism behind the effect of diverse insect pests on the provisioning ecosystem service, that is yield, is not necessarily complementarity (niche differentiation, i.e. additive effects of pests on yield). Complementarity is one of the common hypotheses explaining how species biodiversity affects ecosystem functioning, but it is almost exlcusively tested on organisms providing ecosystem services, rather than disservices. Furthermore, our study shows that to produce satisfactory harvests with reduced inputs of pesticides, we need to take into account all potentially harmful species and the natural variations in the plant damages they cause. Considering the effect of only one pest species separately from other harmful species, may result in economically suboptimal choices being made. Seed yield more than doubled when the level of attack by both seed and stem weevils were high. These results show that increasing our knowledge about multiple pest**–**plant interactions has a large potential for not only directly increasing yield, but also for reducing pesticide input and thereby the negative impacts of agricultural intensification on the environment. However, our study is observational and experiments need to be undertaken before recommendations on crop management can be given. Additionally, it should be noted that the joint effect of pests on yield is probably not fixed, but rather can shift depending on a number of biotic and environmental drivers (e.g. resource availability, competition among plants, plant genetic variation, the strength of pest attack, plant and insect phenology). To quantify the economic consequences of insect attacks, we need to better understand the ecological processes and mechanisms explaining the interactive insect effects on yield, and the scale (plant vs. field) at which they occur. Nevertheless, our results show a promising way that explores a new area of research in agroecology, connects to similar evolutionary studies and has a large potential to lead to a more sustainable agricultural management and increased production.

## Data Accessibility

Data available from the Dryad Digital Repository: http://dx.doi.org/10.5061/dryad.8g3j2

## Conflict of Interest

None declared.

## Supporting information


**Figure S1.** Relationship between seed yield and a) weed cover, b) number of plants per square meter, c) oilseed rape variety and d) amount of nitrogen fertilizers applied in 15 oilseed rape fields.
**Figure S2.** Relationship between main stem damage and main stem height and diameter.
**Figure S3.** Relationship between stem and seed weevil damage.

**Table S1.** Model selection table (coefficients, *df*, log‐likelihood, the value of the information criterion used (AICc), Δ_AIC and ‘Akaike weight') for seed yield. There are 13 alternative models including intercept only model. “NA” – parameter not tested in the model.
