# Supplementary material for: Interactive effects of pests increase seed yield
Source: Ecol Evol. 2016 Feb 29;6(7):2149–57. doi: 10.1002/ece3.2003 (PMC4831447; doi:10.1002/ece3.2003)

**Appendix Fig A.1.** Relationship between seed yield and a) weed cover, b) number of plants per square meter, c) oilseed rape variety and d) amount of nitrogen fertilizers applied in 15 oilseed rape fields. The data for Variety and N fertilizers are obtained from farmers and are missing from 6 and 5 fields, respectively. Weed cover and plant density was estimated using 1m^2^ quadrats per plot (10 samples per field). Weed cover was estimated using categories: 1. <1%, 2. 1-5%, 3. 5-12.5%, 4. 12.5-25%, 5. 25-50%, 6. >50%.

**Appendix Fig A.2.** Relationship between main stem damage and main stem height and diameter.

**Appendix Fig A.3.** Relationship between stem and seed weevil damage.


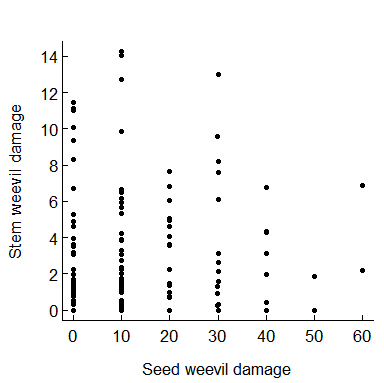

Supplement: Supplementary file 1 — Figure S1. Relationship between seed yield and a) weed cover, b) number of plants per square meter, c) oilseed rape variety and d) amount of nitrogen fertilizers applied in 15 oilseed rape fields. Figure S2. Relationship between main stem damage and main stem height and diameter. Figure S3. Relationship between stem and seed weevil damage. [file ECE3-6-2149-s001.docx]
