# Supplementary material for: Interactive effects of pests increase seed yield
Source: Ecol Evol. 2016 Feb 29;6(7):2149–57. doi: 10.1002/ece3.2003 (PMC4831447; doi:10.1002/ece3.2003)
Supplement: Supplementary file 2 — Table S1. Model selection table (coefficients, df, log‐likelihood, the value of the information criterion used (AICc), Δ_AIC and ‘Akaike weight') for seed yield. There are 13 alternative models including intercept only model. “NA” – parameter not tested in the model. [file ECE3-6-2149-s002.docx]

Appendix Table A.3.

Model selection table (coefficients, df, log-likelihood, the value of the information criterion used (AICc), Δ_AIC and ‘Akaike weight’) for seed yield. There are 13 alternative models including intercept only model. “NA” – parameter not tested in the model.

| (Intercept) | Percentage pollen beetle damage | Percentage stem weevil damage | Percentage seed weevil damage | stem weevil:seed weevil damage | Percentage pod midge damage | pod midge:seed weevil damage | stem weevil:pod midge damage | pollen beetle:pod midge damage | pollen beetle: seed weevil damage | pollen beetle:stem weevil damage | df | logLik | AICc | delta | weight |
| --- | --- | --- | --- | --- | --- | --- | --- | --- | --- | --- | --- | --- | --- | --- | --- |
| 4.545161553 | -0.921078049 | 0.102781364 | 0.065491046 | 0.423124263 | NA | NA | NA | NA | NA | NA | 10 | -211.787 | 445.168 | 0 | 0.875248 |
| 4.544585873 | -0.932968933 | NA | NA | NA | -0.275513814 | NA | NA | 0.150110893 | NA | NA | 9 | -216.688 | 452.67 | 7.501966 | 0.020564 |
| 4.602015137 | -1.0144083 | 0.257013807 | NA | NA | -0.348592981 | NA | NA | NA | NA | NA | 9 | -216.702 | 452.6982 | 7.530152 | 0.020276 |
| 4.53758223 | -0.986377133 | 0.175470279 | NA | NA | -0.323354106 | NA | 0.155814371 | NA | NA | NA | 10 | -215.76 | 453.1144 | 7.946319 | 0.016467 |
| 4.562110337 | -0.947871189 | NA | NA | NA | -0.284492462 | NA | NA | NA | NA | NA | 8 | -218.086 | 453.201 | 8.032987 | 0.015768 |
| 4.499564695 | -0.951365224 | 0.059315854 | NA | NA | NA | NA | NA | NA | NA | 0.232478134 | 9 | -217.132 | 453.5588 | 8.390771 | 0.013186 |
| 4.559710795 | -0.946978918 | NA | 0.09651595 | NA | -0.328082496 | NA | NA | NA | NA | NA | 9 | -217.469 | 454.2331 | 9.065017 | 0.009412 |
| 4.571076429 | -0.902041531 | NA | NA | NA | NA | NA | NA | NA | NA | NA | 7 | -219.878 | 454.5499 | 9.381819 | 0.008033 |
| 4.558881542 | -0.927731225 | NA | -0.002546227 | NA | NA | NA | NA | NA | 0.145180356 | NA | 9 | -217.817 | 454.9282 | 9.760179 | 0.006649 |
| 4.511774496 | -0.928256103 | NA | 0.073662511 | NA | -0.385034771 | 0.07959695 | NA | NA | NA | NA | 10 | -216.807 | 455.2088 | 10.0408 | 0.005778 |
| 4.592996183 | -0.928715856 | 0.156268919 | NA | NA | NA | NA | NA | NA | NA | NA | 8 | -219.377 | 455.7818 | 10.61374 | 0.004339 |
| 4.570701712 | -0.899726124 | NA | 0.02303852 | NA | NA | NA | NA | NA | NA | NA | 8 | -219.801 | 456.6314 | 11.46331 | 0.002837 |
| 4.592136219 | -0.927087182 | 0.153846346 | 0.01360288 | NA | NA | NA | NA | NA | NA | NA | 9 | -219.344 | 457.9829 | 12.81485 | 0.001444 |

Model selection table (coefficients, df, log-likelihood, the value of the information criterion used (AICc), Δ_AIC and ‘Akaike weight’) for seed weight. There are 13 alternative models including intercept only model. “NA” – parameter not tested in the model.

| (Intercept) | Percentage pollen beetle damage | Percentage stem weevil damage | Percentage seed weevil damage | stem weevil:seed weevil damage | Percentage pod midge damage | pod midge:seed weevil damage | stem weevil:pod midge damage | pollen beetle:pod midge damage | pollen beetle: seed weevil damage | pollen beetle:stem weevil damage | df | logLik | AICc | delta | weight |
| --- | --- | --- | --- | --- | --- | --- | --- | --- | --- | --- | --- | --- | --- | --- | --- |
| 0.573477 | 0.004523 | NA | NA | NA | NA | NA | NA | NA | NA | NA | 7 | 154.5663 | -294.338 | 0 | 0.207695 |
| 0.575361 | 0.001457 | 0.014598 | NA | NA | NA | NA | NA | NA | NA | NA | 8 | 155.4783 | -293.928 | 0.410173 | 0.169184 |
| 0.575278 | 0.000303 | 0.018454 | NA | NA | -0.01257 | NA | NA | NA | NA | NA | 9 | 156.1397 | -292.984 | 1.353784 | 0.105549 |
| 0.57322 | 0.004163 | NA | -0.00637 | NA | NA | NA | NA | NA | NA | NA | 8 | 154.8526 | -292.677 | 1.661537 | 0.090496 |
| 0.572957 | 0.004184 | NA | NA | NA | -0.0083 | NA | NA | NA | NA | NA | 8 | 154.8512 | -292.674 | 1.664413 | 0.090366 |
| 0.575309 | 0.001019 | 0.015292 | -0.00678 | NA | NA | NA | NA | NA | NA | NA | 9 | 155.8144 | -292.334 | 2.004357 | 0.076241 |
| 0.574071 | 0.001438 | 0.013305 | -0.00468 | 0.012489 | NA | NA | NA | NA | NA | NA | 10 | 156.6885 | -291.783 | 2.555363 | 0.057881 |
| 0.57503 | 0.001395 | 0.014484 | NA | NA | NA | NA | NA | NA | NA | 0.000963 | 9 | 155.4821 | -291.669 | 2.668876 | 0.054687 |
| 0.57331 | 0.004161 | NA | -0.0049 | NA | NA | NA | NA | NA | 0.006604 | NA | 9 | 155.0842 | -290.874 | 3.464711 | 0.036734 |
| 0.574137 | 0.000532 | 0.017022 | NA | NA | -0.01214 | NA | 0.003173 | NA | NA | NA | 10 | 156.1896 | -290.785 | 3.553309 | 0.035143 |
| 0.572872 | 0.003835 | NA | -0.00511 | NA | -0.00669 | NA | NA | NA | NA | NA | 9 | 155.0218 | -290.749 | 3.589533 | 0.034512 |
| 0.572768 | 0.004365 | NA | NA | NA | -0.00716 | NA | NA | 0.003807 | NA | NA | 9 | 154.8988 | -290.503 | 3.835598 | 0.030517 |
| 0.573324 | 0.003808 | NA | -0.00501 | NA | -0.00606 | -0.00094 | NA | NA | NA | NA | 10 | 155.0276 | -288.461 | 5.877199 | 0.010995 |

Model selection table (coefficients, df, log-likelihood, the value of the information criterion used (AICc), Δ_AIC and ‘Akaike weight’) for seed number. There are 13 alternative models including intercept only model. “NA” – parameter not tested in the model.

| (Intercept) | Percentage pollen beetle damage | Percentage stem weevil damage | Percentage seed weevil damage | stem weevil:seed weevil damage | Percentage pod midge damage | pod midge:seed weevil damage | stem weevil:pod midge damage | pollen beetle:pod midge damage | pollen beetle: seed weevil damage | pollen beetle:stem weevil damage | df | logLik | AICc | delta | weight |
| --- | --- | --- | --- | --- | --- | --- | --- | --- | --- | --- | --- | --- | --- | --- | --- |
| 26.00678 | -0.24825 | NA | 1.004871 | NA | NA | NA | NA | NA | NA | NA | 8 | -378.372 | 773.7717 | 0 | 0.282733 |
| 25.73161 | -0.28592 | NA | 0.94461 | NA | -0.65164 | 0.537182 | NA | NA | NA | NA | 10 | -376.372 | 774.3377 | 0.56601 | 0.213044 |
| 25.99851 | -0.32789 | NA | 1.059588 | NA | -0.46757 | NA | NA | NA | NA | NA | 9 | -377.544 | 774.3829 | 0.611282 | 0.208276 |
| 26.00715 | -0.23526 | NA | 0.99626 | NA | NA | NA | NA | NA | -0.16182 | NA | 9 | -378.101 | 775.4965 | 1.72481 | 0.119355 |
| 25.98174 | -0.2141 | -0.222 | 1.01145 | NA | NA | NA | NA | NA | NA | NA | 9 | -378.167 | 775.6284 | 1.85673 | 0.111736 |
| 25.972 | -0.21247 | -0.23647 | 1.051167 | 0.344863 | NA | NA | NA | NA | NA | NA | 10 | -377.629 | 776.8528 | 3.08116 | 0.060578 |
| 26.02914 | -0.22566 | NA | NA | NA | NA | NA | NA | NA | NA | NA | 7 | -384.508 | 783.81 | 10.03839 | 0.001869 |
| 26.02406 | -0.26655 | NA | NA | NA | -0.24002 | NA | NA | NA | NA | NA | 8 | -384.297 | 785.623 | 11.85137 | 0.000755 |
| 26.00495 | -0.1889 | -0.20561 | NA | NA | NA | NA | NA | NA | NA | NA | 8 | -384.368 | 785.7639 | 11.99226 | 0.000704 |
| 25.90169 | -0.2144 | -0.27095 | NA | NA | NA | NA | NA | NA | NA | 0.246968 | 9 | -384.152 | 787.5985 | 13.82683 | 0.000281 |
| 26.00272 | -0.23007 | -0.18041 | NA | NA | -0.21931 | NA | NA | NA | NA | NA | 9 | -384.195 | 787.6852 | 13.9135 | 0.000269 |
| 26.03456 | -0.27767 | NA | NA | NA | -0.26908 | NA | NA | -0.11676 | NA | NA | 9 | -384.239 | 787.7733 | 14.0016 | 0.000258 |
| 26.06874 | -0.26267 | -0.05811 | NA | NA | -0.25385 | NA | -0.22742 | NA | NA | NA | 10 | -383.672 | 788.9377 | 15.16605 | 0.000144 |
